# Dataset supporting the estimation and analysis of high spatial resolution inventories of atmospheric emissions from several sectors in Argentina

**DOI:** 10.1016/j.dib.2020.105281

**Published:** 2020-02-13

**Authors:** Salvador Enrique Puliafito, Tomás Rafael Bolaño-Ortiz, Lucas Luciano Berná Peña, Romina María Pascual-Flores

**Affiliations:** a GEAA - Grupo de Estudios Atmosféricos y Ambientales, UTN-FRM - Universidad Tecnológica Nacional - Facultad Regional Mendoza, Mendoza, Argentina; b CONICET - Consejo Nacional de Investigaciones Científicas y Técnicas, Buenos Aires, Argentina; c ANPCyT - Agencia Nacional de Promoción Científica y Tecnológica, Buenos Aires, Argentina

**Keywords:** Air quality model, High-resolution emissions inventory, Greenhouse gas, Methane, Energy, Livestock production, Agriculture, Biomass burning

## Abstract

This data article provides an extensive and complete description of the high spatial resolution inventory (HSRI) estimation shown in the article “High resolution inventory of atmospheric emissions from livestock production, agriculture, and biomass burning sectors of Argentina” Puliafito et al. [1], and its comparison with several sectors in Argentina. The dataset provided are high-resolution inventories (0.025° × 0.025° lat/long) for CO_2_, CH_4_, N_2_O and another 8 species from livestock, biomass burning, agriculture and another 12 sectors (based on 2016 year). In addition, we also provide the database for 2014 using the same methodology. The dataset presented are necessary to improve input inventories for air quality models. Also, they are better to inform and guide the stakeholders, in making decisions related to environmental protection and health promotion, as well as assessing the environmental performance in terms of atmospheric emissions of an activity, sector or region in Argentina.

**Table:** Specifications Table

Subject	Environmental Science, Pollution
Specific subject area	Estimation of high spatial resolution inventory from livestock production, agriculture, and biomass burning sectors
Type of data	Tables and figures
How data were acquired	Data collection of the amount and type of production for each sector, and estimation through different methods. In addition, specific emission factors were applied for each sector for the calculation of the different polluting species. [[1], [2], [3]]
Data format	Raw and Processed
Parameters for data collection	The data were estimated for the activity level recorded in 2016 and 2014 for each sector and polluting species.
Data source location	Country: Argentina Latitude and longitude: 22° to 56°S latitude and from 52° to 75°W longitude
Data accessibility	With the article and a public repository: Repository name: Mendeley Data Data identification number: 10.17632/nj5yf7k3s7.1 Direct URL to data: https://doi.org/10.17632/nj5yf7k3s7.1
Related research article	Puliafito S. E., Bolaño-Ortiz T.R., Berná L.L., Pascual-Flores, R.M. (2020) High resolution inventory of atmospheric emissions from livestock production, agriculture, and biomass burning sectors of Argentina, Atmos. Environ*.*https://doi.org/10.1016/j.atmosenv.2019.117248 [1]

**Table:** 

**Value of the Data** • The dataset improves input inventories for air quality models with high spatial resolution in Argentina. • The dataset increases knowledge related to a spatial distribution of atmospheric emissions in Argentina, because it includes detailed species emitted from different sectors. • The dataset informs and guides the stakeholders, in making better decisions related to environmental protection and health promotion. • The dataset can be compared with the dataset of same sector/species from different regions/countries. Also, they improve the spatial resolution from global inventories for Argentina.

## 1 Data

Dataset reported in this article concerns the annual atmospheric environmental emissions generated by 15 sectors (based on years 2014 and 2016). This inventory has 711,554 cells that include the continental and maritime zone of Argentina for the geographical frame located within a rectangular box from 22°S to 56°S latitude and from 52°W to 75°W longitude for each sector and species respectively. Table 1 shows the sectors and pollution species considered in this data article. The annual amounts for the years considered for each sector and pollutants of Argentina [1] are shown in Table 2. Dataset is available in detail for a high resolution (0.025° × 0.025° lat/long) in all sectors and their species respectively (see the repository).

**Table 1:** The sectors (rows) and polluting species (columns) considered.

	BC	SO_2_	N_2_O	PM_10_	PM_2.5_	NH_3_	NMVOC	NO_X_	CO	CH_4_	CO_2_
Airplanes	●	●	●	●	●		●	●	●	●	●
Ships	●	●	●	●	●		●	●	●	●	●
Trains	●	●	●	●	●	●	●	●	●	●	●
Road transport	●	●	●	●	●	●	●	●	●	●	●
Cement industry	●	●		●	●		●	●	●		●
Oil production refineries	●	●	●	●	●		●	●	●	●	●
Fugitives, venting, flares	●	●	●	●	●	●	●	●	●	●	●
Power plants	●	●	●	●	●		●	●	●	●	●
Residential	●	●	●	●	●		●	●	●	●	●
Commercial	●	●	●	●	●		●	●		●	●
Livestock production	●		●	●	●	●	●	●	●	●	
Agriculture			●	●	●	●	●	●		●	●
Biomass burning	●	●	●	●	●	●	●	●	●	●	●
Urban waste										●	
Industry	●	●	●	●	●	●	●	●	●	●	●

**Table 2:** Total amount of atmospheric emissions (Gg) for various sectors (rows) and polluting species for each sector (columns).

	BC		SO_2_		N_2_O		PM_10_		PM_2.5_		NH_3_		NMVOC		NO_X_		CO		CH_4_		CO_2_	
	2014	2016	2014	2016	2014	2016	2014	2016	2014	2016	2014	2016	2014	2016	2014	2016	2014	2016	2014	2016	2014	2016
Airplanes	0.004	0.004	0.697	0.720	0.071	0.074	0.057	0.074	0.071	0.059	0.000	0.000	0.382	0.394	9.925	10.242	6.416	6.621	0.059	0.061	2223.906	2295.071
Ships	0.099	0.103	8.055	8.377	0.805	0.838	0.916	1.748	1.681	0.952	0.000	0.000	1.105	1.149	31.799	33.071	2.980	3.100	2.819	2.932	2530.383	2631.599
Trains	0.067	0.072	0.021	0.022	0.002	0.002	0.062	0.116	0.109	0.066	0.001	0.001	0.350	0.378	3.948	4.264	0.806	0.869	0.014	0.015	236.605	255.534
Road transport	0.522	0.551	12.626	13.255	3.561	3.736	5.797	10.142	9.661	6.087	3.259	3.419	368.533	386.959	436.297	458.111	1740.469	1827.492	16.518	17.342	48581.586	51010.666
Cement industry	0.240	0.229	3.029	2.892	0.000	0.000	0.377	0.624	0.654	0.360	0.000	0.000	0.150	0.143	10.063	9.610	11.823	11.291	0.000	0.000	4134.000	3947.970
Oil production refineries	0.004	0.004	0.253	0.269	0.008	0.008	0.034	0.068	0.065	0.036	0.000	0.000	0.344	0.365	10.401	11.025	1.370	1.452	0.072	0.076	3962.433	4200.179
Fugitives, venting, flares	17.704	18.589	0.027	0.028	0.023	0.024	44.336	77.727	74.021	46.554	0.028	0.030	77.721	81.814	41.272	43.366	2.328	2.491	272.135	293.553	3488.947	3663.394
Power plants	0.150	0.158	49.407	51.927	1.208	1.270	2.362	4.006	3.811	2.921	0.000	0.000	6.352	6.676	158.681	166.774	29.458	30.960	4.582	4.815	40329.848	42386.670
Residential	0.442	0.458	1.327	1.373	0.118	0.121	7.585	13.084	12.642	7.066	0.000	0.000	5.869	6.074	64.231	66.479	101.305	104.851	3.633	3.760	25678.576	26577.326
Commercial	0.026	0.027	0.419	0.433	0.045	0.046	0.450	0.777	0.751	0.420	0.000	0.000	0.496	0.513	13.015	13.470	8.604	8.905	0.140	0.145	5214.334	5396.836
Livestock production	0.390	0.392	0.000	0.000	79.444	82.645	11.136	26.270	23.862	11.024	180.507	189.604	294.180	199.071	6.845	6.915	0.000	0.000	2647.307	2679.129	0.000	0.000
Agriculture	0.000	0.000	0.000	0.000	34.036	33.163	1.135	76.137	2.064	8.969	159.478	101.714	13.758	17.473	10.317	8.254	0.000	0.000	32.522	48.429	349.373	2927.744
Biomass burning	2.854	2.825	1.282	1.475	0.629	0.763	52.847	104.326	15.639	52.364	6.155	8.065	5.649	7.662	5.521	14.850	498.396	590.782	112.289	33.357	6431.658	9517.696
Urban waste	0.000	0.000	0.000	0.000	0.000	0.244	0.000	0.000	0.000	0.000	0.000	0.000	0.000	0.000	0.000	0.000	0.000	0.000	581.352	120.340	0.000	30.201
Industry	4.480	4.615	34.586	35.623	0.463	0.477	45.633	85.458	82.969	47.002	3.694	3.805	13.967	14.386	57.379	59.100	380.301	391.710	3.335	3.435	21637.268	22286.386

## 2 Experimental design, materials, and methods

Data on HSRI for various Argentinians sectors management procedures was collected through primary and secondary sources of local accessible reports, literature and guidelines. The global methodology applied is based on the European regulations grouped on European Monitoring and Evaluation Program (EMEP) [4,5]. In addition, the used emission factors are those indicated by EMEP according to the adopted level of detail, which concerns level 2 in almost all cases. In some cases, the data on the activity and the use of specific national emission factors were used following the Third National Communication (TCNA) [6] of Argentina to the United Nations Convention on Climate Change. For specific subsectors, we used the recommendations (which are indicated in each case) of the Intergovernmental Panel on Climate Change (IPCC) [7]. With respect to the inventory of Livestock, Agriculture, and Biomass burning sectors they have been provided by Ref. [1]. Road transport sector was updated using the same resolution of this article following the steps from Ref. [3] and the other sectors by Refs. [2,8].
